# Knowledge, Attitudes, and Practices Toward Coronavirus and Associated Anxiety Symptoms Among University Students: A Cross-Sectional Study During the Early Stages of the COVID-19 Pandemic in Bangladesh

**DOI:** 10.3389/fpsyt.2022.856202

**Published:** 2022-04-01

**Authors:** Muhammad Mainuddin Patwary, Asma Safia Disha, Mondira Bardhan, Md. Zahidul Haque, Md. Pervez Kabir, Sharif Mutasim Billah, Md. Riad Hossain, Md. Ashraful Alam, Matthew H. E. M. Browning, Faysal Kabir Shuvo, Awais Piracha, Bo Zhao, Sarya Swed, Jaffer Shah, Sheikh Shoib

**Affiliations:** ^1^Environment and Sustainability Research Initiative, Khulna, Bangladesh; ^2^Environmental Science Discipline, Life Science School, Khulna University, Khulna, Bangladesh; ^3^Institute of Disaster Management, Khulna University of Engineering & Technology, Khulna, Bangladesh; ^4^Department of Global Health Policy, Graduate School of Medicine, The University of Tokyo, Tokyo, Japan; ^5^Department of Parks, Recreation and Tourism Management, Clemson University, Clemson, SC, United States; ^6^Centre for Urban Transitions, Swinburne University of Technology, Melbourne, VIC, Australia; ^7^Geography, Tourism and Urban Planning, Western Sydney University, Penrith, NSW, Australia; ^8^Department of Health Administration, Graduate School, Yonsei University, Wonju, South Korea; ^9^Faculty of Human Medicine, Aleppo University, Aleppo, Syria; ^10^Medical Research Center, Kateb University, Kabul, Afghanistan; ^11^Department of Psychiatry, Jawahar Lal Nehru Memorial Hospital (JLNMH), Srinagar, India

**Keywords:** KAP, anxiety, COVID-19, cross-sectional, knowledge, university student, Bangladesh

## Abstract

**Background:**

University students’ knowledge, attitude, and practice (KAP) toward COVID-19 are vital to prevent the spread of the virus, especially in the context of developing countries. Consequently, the present study aimed to determine the KAP levels of university students and associated anxiety during the earlier stage of the pandemic in Bangladesh.

**Methods:**

A cross-sectional, online study with 544 university students was conducted during April 17–May 1, 2020. The questionnaire incorporated several KAP-related test items aligned with the World Health Organization (WHO) guidelines. Anxiety was measured with the 2-item Generalized Anxiety Disorder scale (GAD-2). Multivariable logistic regression analysis was performed to determine the association between KAP levels and anxiety adjusting for sociodemographic variables. Subgroup analyses included rerunning models stratified by gender and quarantine status.

**Results:**

Approximately 50% of students showed high levels of knowledge about COVID-19 guidelines, 59% reported behavioral practices that aligned with COVID-19 guidelines, and 39% had negative attitudes toward COVID-19 guidelines. Attitudes differed by anxiety (χ^2^ = 23.55, *p* < 0.001); specifically, negative attitudes were associated with higher anxiety (OR: 2.40, 95% CI = 1.66–3.46, *p* < 0.001). Associations were significant for male (OR = 2.36; 95% CI = 1.45–3.84, *p* < 0.001) and female (OR = 2.45; 95% CI = 1.3–4.34; *p* < 0.001) students. Stratified analyses found non-quarantined students with negative attitudes had three times the chance of experiencing anxiety (OR = 3.14, 95% CI: 1.98–4.98, *p* < 0.001). Non-quarantined students with low levels of knowledge had half the chance of developing anxiety (OR = 0.49, 95% CI: 0.31–0.78, *p* < 0.01).

**Conclusion:**

Based on these findings, it is recommended that university authorities continue to prioritize proactive and effective measures to develop higher levels of knowledge, more positive attitudes and better behavioral practices regarding COVID-19 for the mental health of their students.

## Introduction

The rapidly spreading coronavirus disease 2019 (COVID-19) has been recognized as a worldwide public health concern. The World Health Organization (WHO) declared COVID-19 a public health emergency of international concern on 30 January 2020 and urged all nations to work together to halt the epidemic (1). In response, countries around the world implemented a variety of containment measures, including the closure of educational and other government and non-government institutions, prohibition of large-scale social gatherings, restrictions on local, national, and international travel, and complete lockdowns to prevent viral transmission (2, 3). Despite these precautions, the world has recorded a massive number of infected cases, about 262 million, with 5.2 million deaths to-date because of the highly contagious nature of the coronavirus (4).

The first COVID-19 case in Bangladesh was reported on March 08, 2020 (5). As one of the most densely populated countries, Bangladesh faced particularly demanding challenges to manage the knowledge, attitudes, and behavior practices regarding COVID-19 in its massive population (6, 7). As of August 14, 2021, Bangladesh reported COVID-19 cases surpassed 1.4 million, and COVID-19-related deaths exceeded 23,600 (5). To control the spread of the virus, the Bangladesh government has taken several precautionary measures, including educational institution shutdowns, ceasing all social gatherings, closing government and non-government entities except emergency services, restricting tourism, and limiting intra-country travel (7–9). Besides, several organizations voluntarily promoted massive advertisements regarding COVID-19 on awareness-raising, proper handwashing practices, wearing facemask appropriately, and maintaining social distancing, among other measures (10).

For Bangladesh to further control the virus, each citizen must be informed, maintain attitudes that support adherence to behavioral practices, and practice measures that reduce health risks and viral transmission (11). Therefore, appropriate knowledge, attitude, and practice (KAP) levels toward this infectious disease are cognitive keys to this public health emergency (12). KAP entails a variety of ideas regarding the disease’s etiology and exacerbating variables, and the identification of symptoms, treatment options, and repercussions (13). Studies during the Severe Acute Respiratory Syndrome (SARS) pandemic showed poor level of KAP concerning contagious diseases was an obstacle to containment (14). During the COVID-19 pandemic, some scholars believe that poor knowledge and orthodox religious beliefs may be responsible for negative attitudes and ineffective containment strategies (6).

Knowledge, attitude, and practice surrounding health-related habits, along with environmental and financial factors, status of quarantine, lockdown measures and fear of COVID-19 may influence anxiety levels during the pandemic (15, 16). According to a previous study on Brazilian people, respondents experienced fear and mental distress due to multi-level coping strategies (17). Another study conducted in Latvia found that poor health conditions, fear of contracting COVID-19, having family members contract COVID-19, family conflicts, lack of religiosity and caring for a vulnerable person were associated with depression and anxiety (18). Several studies on infectious diseases found that knowledge and attitude toward these diseases were related to serious psychological distress, fear, and stigma among people that challenged efforts to prevent disease spread (19–21). In the 2003 SARS outbreak, lower levels of anxiety were associated with higher levels of knowledge and positive attitudes toward infectious disease transmission (22). A cross-sectional study of Chinese college students during COVID-19 found that knowledge and attitudes were protective against mental distress (21). Another study in Latvia reported that preventive behaviors during COVID-19 were associated with COVID-19 threat appraisal, trust in information sources, and fear (23). Another study identified protective factors of COVID-19 including disbelief in the effectiveness of precautionary behavior were associated with lockdown-induced anxiety (24). A global analysis of 40 countries reported that physical inactivity, excessive use of the internet, tendency to stay up late, sleeping pills and dreams of being trapped contributed to anxiety during the COVID-19 lockdown (25). Ding et al. (26) conducted a study of 817 pregnant women and reported that high knowledge scores were associated with less anxiety. Alaloul et al. (27) reported high levels of anxiety were associated with preventive measures in Oman during the pandemic. Other studies in Singapore, China and Italy found that self-efficacy and information sufficiency was associated with lower anxiety levels, while higher anxiety levels were catalysts to adopt preventive behaviors (28). Another study in Indonesia found that individuals with correct responses to knowledge tests had significantly lower anxiety scores. That study also found individuals reporting practices that conflicted with WHO guidance, such as attending crowded places, showed higher anxiety scores (29). Collectively, these studies suggest KAP level are associated with anxiety during the pandemic. However, some contradictory evidence also exists; high anxiety levels in India (30) and China (31) were found even in respondents with reasonably good levels of knowledge about the virus. Chowdhury et al. (32) reported anxiety due to COVID-19 was negatively associated with risky behavior during COVID-19 outbreaks. Based on this literature and the emerging COVID-19 situation, it remains important to determine level of KAP surrounding the coronavirus and its associations with anxiety. Such knowledge would provide further insight into how Bangladesh can prevent the further spread of the contagion and downstream impacts of its citizens’ mental health.

At present in Bangladesh, the literacy rate of current status stands for 74.9%, while the Net Enrollment Rate (NER) on primary education is almost 97.94% and over 1.3 million students receive tertiary level of education, of which 74% were male, and 26% were female (33, 34). In the present study, we studied KAP surrounding COVID-19 and anxiety among university students. This sample was chosen because we expected they would be motivated and insightful regarding positive attitudes toward COVID-19 containment measures. In Bangladesh, there have been several studies on the KAP of students and young adults (35–39). Further, Hossain and his research team investigated Bangladeshi general people’s KAP toward COVID-19 and their underlying fear levels in relation to sociodemographic factors (40). However, the KAP of university students and their association with mental distress has yet to be investigated. Based on this research gap, the present study aimed to determine KAP toward COVID-19 and associated anxiety of university students during the earlier stage of the pandemic in Bangladesh. More specifically, this study aimed to:

a. Determine Knowledge, Attitude, and Practice (KAP) levels of university students toward COVID-19.
b. Test associations between KAP levels and anxiety during the COVID-19 lockdown period.
c. Explore gender-based differences in associations between KAP levels and anxiety.
d. Examine quarantine status-based differences in KAP levels and anxiety associations.

## Materials and Methods

### Study Participants and Sampling Procedure

A cross-sectional study was conducted during the first wave of the pandemic among university students of Bangladesh to understand their KAP levels and anxiety during the COVID-19 pandemic. Inclusion criteria included current enrollment as a university student and the decision to participate in our study. The questionnaire was prepared in English and then translated to the local language (Bangla). A snowball sampling procedure was used to collect the data. First, we distributed a web-based structured questionnaire through attainable social networks (e.g., Facebook, WhatsApp, and Instagram). Then, we requested our social network communities to provide their responses and asked them to share the questionnaire with their networks. The questionnaire was prepared following the World Health Organization (1) guidelines. It was divided into the following sections: (a) demographic information, (b) knowledge toward the COVID-19 pandemic, (c) attitudes toward the spread of COVID-19, (d) related practices to control the spread of COVID-19, and (e) anxiety.

The questionnaire’s relevance was determined by consulting a panel of experts. A relevance analysis was used to determine the content validity of each questionnaire block. Experts offered constructive feedback on readability, general relevance, and specific relevance to the study’s aims. The questionnaire was then piloted with 25 participants to gather additional feedback. The questionnaire was modified based on this feedback and made more understandable. Cronbach’s alpha values were used to determine the reliability index of both the pilot and final questionnaires. All values were more than 0.75, suggesting that the reliability was satisfactory (41).

Previous studies using our suite of measures were unavailable; therefore, we used an online calculator to estimate our necessary sample size (42). We followed the recommended conservative value (50%) for the proportion of our sample displaying our factor of interest. Thus, we calculated the minimum required number of respondents using an online sample size calculator,^1^ which was determined at 427 based on a 10% non-response rate, 5% precision, and 50% proportion, with a 95% confidence range for the overall population size of 3.2 million of tertiary level students in Bangladesh (43).

We gathered 744 responses between April 17 and May 01, 2020. As our target population was university students, we cross-checked our data and found that among the respondents, 544 students from different universities of Bangladesh responded. Thus, a total of 544 responses were used for the final analysis. All survey items were answered by all participants, so missing data analysis was not required. Electronic consent was obtained from all participants prior to their completion of the survey. The participant could opt out at any time. Additionally, the survey did not ask participants to provide their names or email addresses, ensuring that the participant could not be identified. Accordingly, the research ethical clearance board of the Institute of Disaster Management, Khulna University of Engineering & Technology, Khulna, Bangladesh waived the approval for this study.

### Measures

The survey gathered information on the independent (KAP levels) and dependent variable (anxiety) as well as basic information on university students. This basic information included their gender, age, degree of education, residential status, living status, quarantine status, and sources of information during the COVID-19 epidemic.

• Participants’ knowledge of the COVID-19 pandemic was assessed using 24 yes/no questions about the illness type, mode of transmission, and likelihood of exposure to transmission risk. Respondents were asked to answer questions as true or false, with the option of “don’t know.” Correct responses received a score of one, while incorrect or unsure (don’t know) responses received a score of zero. The overall score for knowledge was between 0 and 24. Scores greater than the sample mean were classified as having good knowledge while scores having less than the sample mean were classified as having poor knowledge. This differentiation between good and poor knowledge levels is in line with past research, improved the interpretability of the results, and was responsive to differences in information sources between populations (44). Cronbach’s alpha value of 0.78 suggested a high degree of internal consistency.
• Attitudes toward COVID-19 were captured with five questions divided into two categories. Three questions captured negative attitudes toward COVID-19: (1) worry about the personal financial condition, (2) worry about academic delays, and (3) worry about social stigma. Two items captured positive attitudes toward COVID-19: (4) daily life returning to normal soon, and (5) social support during the pandemic. To determine the extent of these attitudes, a five-point Likert-type scale was used with responses ranging from 1 (not at all) to 5 (very high). The total attitude score ranged from 5 to 25, with negative attitude items recoded to align with the directionality of the response scale of the positive attitude items. Individuals who scored higher on the attitude scale than the mean were categorized as having a positive attitude, while those who scored lower than the mean on the attitude scale were labeled as having a negative attitude. This categorization also aligned with past research, assisted with the interpretability of the results, and was responsive to population differences (44). The Cronbach’s alpha was 0.80, indicating a high degree of internal consistency.
• To capture preventive measures, respondents were asked ten questions about their precautionary behaviors during the COVID-19 pandemic. Each item was answered as 1 (yes) or 0 (no). The total score ranged from 0 to 10. Once again, this score was classified into two levels for the same reasons as the knowledge and attitude classifications (44). A score higher than the mean indicated good practices and a score less than the mean indicated poor practices.
• Anxiety was assessed with the 2-item Generalized Anxiety Disorder scale (GAD-2). GAD-2 is a shortened version of the GAD-7 that has been reported in numerous studies to assess anxiety disorders (45, 46). The GAD-2 evaluates how participants were bothered over the last 2 weeks by “feeling nervous, anxious, or edge” and “not being able to stop or control worrying” (47). Participants responded on a 4-point Likert scale: 0 (not at all) to 3 (almost every day). The total score ranges from 0 to 6 with scores ≥ 3 indicating a higher level of anxiety (47).

### Statistical Analysis

Descriptive statistics were calculated to analyze the characteristics of the respondents. Levels of KAPs were reported as frequency distributions. Categorical data were presented as numbers (*N*) and frequencies (%), while continuous data were displayed as means and standard deviations (SD). Associations between KAP levels and anxiety were tested with Pearson chi-square tests and Student’s *t*-tests. To determine associations between KAP levels and anxiety while adjusting for other factors, multivariable logistic regression models were run. Controls included gender, age, education, place of residence, living status, quarantine status, and information sources. Stratified analyses were conducted to examine associations between KAP levels and anxiety in men vs. women and in students under vs. outside of quarantine. The significance of associations was determined with odds ratios (OR) and 95% confidence intervals (CI). A two-tailed test with a significance level of *p* < 0.05 was considered statistically significant. The R Statistical Package (version 4.0), developed by R Core Team released on 2021 and IBM SPSS Statistics, Version 26.0. IBM Corp., Armonk, NY, United States were used to analyze the data.

## Results

### Sample Demographics

Demographic characteristics of students are displayed in Table 1. Of the total, 56.99% were men, and 43.01% were women. The majority (72.43%) were 25 years old or less. Most were undergraduate students (66.54%), followed by graduate (27.21%) and post-graduate (6.25%) students. A total of 84.74% were urban residents, and 78.86% lived with family members. Approximately one-third (32.17%) were in quarantine during the survey period. The largest share of respondents used social media to collect information about the pandemic (89.34%), followed by traditional media (77.57%), governmental agencies (77.21%), online media (60.85%), and healthcare staff (31.43%). Females (χ2 = 12.34, *p* < 0.05), participants over the age of 25 (χ2 = 19.32, *p* < 0.05), graduate students (χ2 = 18.34, *p* < 0.05), and non-quarantined students (χ2 = 4.56, *p* < 0.05) were more likely to show high anxiety relative to their counterparts.

**TABLE 1:** Demographic characteristics of respondents for the total sample and among students without vs. with anxiety, and significance tests for differences between each characteristic and anxiety (*N* = 544).

Characteristics	*N* (%)			χ 2	*P*-value^a^
	Total (*N* = 544)	Without anxiety (*N* = 295)	With anxiety (*N* = 249)		
**Gender**				12.34	0.03*
Male	310 (56.99)	180 (61.02)	130 (52.21)		
Female	234 (43.01)	115 (38.98)	119 (47.79)		
**Age**				19.32	0.01*
≤25	394 (72.43)	226 (76.61)	168 (67.46)		
>25	150 (27.57)	69 (23.39)	81 (32.54)		
**Education status**				18.34	0.01*
Undergraduate student	362 (66.54)	210 (71.19)	152 (61.05)		
Graduate student	148 (27.21)	71 (24.07)	77 (30.92)		
Post-graduate student	34 (6.25)	14 (4.75)	20 (8.03)		
**Place of residence**				1.45	0.34
Urban	461 (84.74)	246 (83.39)	215 (86.35)		
Rural	83 (15.26)	49 (16.61)	34 (13.65)		
**Living status**				1.02	0.31
Alone	26 (4.78)	15 (5.08)	11 (4.42)		
With family members	429 (78.86)	236 (80.00)	193 (77.51)		
With non-family members	89 (16.36)	44 (14.92)	45 (18.07)		
**Quarantine status**				4.56	0.04*
Yes	175 (32.17)	94 (31.86)	81 (32.53)		
No	369 (67.83)	201 (68.24)	168 (67.47)		
**Information source for COVID-19**				0.35	0.96
Government agency	420 (77.21)	228 (77.29)	192 (77.11)		
International agency	347 (63.79)	186 (63.05)	161 (64.66)		
Healthcare staff	171 (31.43)	96 (32.54)	75 (30.12)		
Social media	486 (89.34)	257 (87.12)	229 (91.97)		
Traditional media	422 (77.57)	224 (75.93)	198 (79.52)		
Online media	331 (60.85)	177 (60.00)	154 (61.85)		

^a^Kruskal–Wallis Test, *p < 0.05.

### Knowledge, Attitudes, Practices, and Anxiety Levels

Frequencies of correct and incorrect answers to knowledge-related questions are provided in Supplementary Table 1. Almost all students (91.91%) agreed with the statement, “COVID-19 is an infectious disease.” About 94.67% of students answered correctly for droplets as one of the transmission routes of the virus, followed by a face-to-face talk (77.39%), handshaking (97.06%), fecal-oral transmission (66.36%), mosquito bites (72.06%), and touching of objects used by an infected person (96.32%). Most students responded incorrectly that food, air, and pets could transmit COVID-19. More than nine-in-ten respondents knew the common symptoms of COVID-19 such as fever (95.77%), dry cough (90.81%), sore throat (91.91%), and difficulty breathing (93.93%). Most students provided incorrect answers for nose bleeds (95.77%) and aches and pains (60.29%). Another 87.50% of students gave the correct answer for the incubation period as understood at the time of this study (1–14 days). When respondents were asked about individuals at most risk of COVID-19, most students correctly answered that people over 60 years old (97.61%), people with chronic illness (92.28%), healthcare professionals (91.36%), and pregnant women (54.23%) were at increased risk of COVID-19. On the other hand, 71.14% of participants believed that young people were not at high risk of COVID-19. One exception was noticed for children, where more than half provided the incorrect answer.

Supplementary Table 2 shows the positive and negative attitudes students held toward COVID-19 during the lockdown. Nearly half of the respondents believed that life would be back to normal soon. However, 29.41% were not optimistic and reported being undecided about this statement. Approximately 70% believed in the necessity of social support during the pandemic. In contrast, more than half were worried about their economic condition being at risk. Another 39.89% consented that they were worried about their academic routine while almost one-third were undecided about this. Besides, 31.25% were undecided about infected people facing stigma in the society.

Regarding practices toward COVID-19, 56.86% of respondents reported that they were staying at home (Supplementary Table 3). The vast majority did not wash their hands more frequently with soap and water (91.36%) or avoid social gatherings (92.83%) and public transports (86.21%).

Table 2 shows the KAP scores of students with and without anxiety during COVID-19. Of the total, 50.55% (*N* = 275) demonstrated a high level of knowledge, 38.61% showed a negative attitude, and 59.01% maintained good practices regarding the COVID-19 pandemic. Students with anxiety had significantly higher negative attitudes about COVID-19 (52.85%, χ2 = 23.55, *p* < 0.001). There were no statistically significant differences in knowledge and practice scores between students with and without high anxiety.

**TABLE 2:** Knowledge, attitude, and practice (KAP) levels toward COVID-19 among students with and without anxiety, and significant tests between each level and anxiety.

KAP score	*N* (%)			χ 2	*p*-value
	Total	Without anxiety	With anxiety		
Knowledge				1.836	0.192
High	275 (50.55)	157 (57.09)	118 (42.91)		
Low	269 (49.45)	138 (51.30)	131 (48.70)		
Attitudes				23.55	0.000***
Positive	334 (61.39)	186 (55.20)	151 (44.80)		
Negative	210 (38.61)	99 (47.14)	111 (52.85)		
Practices				0.035	0.853
Good	321 (59.01)	173 (53.89)	148 (46.11)		
Bad	223 (40.99)	122 (54.71)	101 (45.29)		

Chi-square test was conducted to identify significance difference. ***p < 0.001 (2-tailed).

### Associations Between Knowledge, Attitude, and Practice Levels and Anxiety

Bivariate correlations between KAP levels and anxiety are reported in Table 3. Knowledge was positively correlated with attitudes (*r* = 0.156, *p* < 0.01) and practices (*r* = 0.227, *p* < 0.01). Attitudes were also positively correlated with practices (*r* = 0.178, *p* < 0.01). Anxiety was positively correlated with attitudes (*r* = 0.287, *p* < 0.01) but not with knowledge or practices (*p* > 0.05).

**TABLE 3:** Correlations between knowledge, attitude, and practice (KAP) levels toward COVID-19 and anxiety among university students in Bangladesh during the early phases of the pandemic (*N* = 544).

	Knowledge	Attitudes	Practices	Anxiety
Knowledge	1			
Attitudes	0.156**	1		
Practices	0.227**	0.178**	1	
Anxiety	0.007	0.287**	0.081	1

**p < 0.01 (2-tailed).

Table 4 presents the results of a logistic regression model used to determine fully adjusted associations between KAP levels and anxiety. After accounting for co-variables (gender, age, education, place of residence, living status, quarantine status, and information sources), participants with negative attitudes toward COVID-19 expressed 2.4 times higher risk of anxiety (95% CI: 1.66–3.46, *p* = 0.000). Associations between practices and anxiety were not statistically significant, whereas associations between knowledge and anxiety were negative and approached significance (OR = 0.71, 95% CI: 0.50–1.02, *p* = 0.06). However, none of adjusted variables were found significant (Supplementary Table 4).

**TABLE 4:** Multivariate logistic regression models to determine associations between knowledge, attitude, and practice (KAP) levels toward COVID-19 and anxiety among university students in Bangladesh during the early phases of the pandemic (*N* = 544).

KAP levels	Crude model		Fully adjusted model^a^	
	OR (95% CI)	*P*-value	OR (95% CI)	*P*-value
**Knowledge**				
Low	0.72 (0.51–1.03)	0.06	0.71 (0.50–1.02)	0.06
High	1.00 (ref.)		1.00 (ref.)	
**Attitudes**				
Negative	2.43 (1.71–3.45)	0.000***	2.40 (1.66–3.46)	0.000***
Positive	1.00 (ref.)		1.00 (ref.)	
**Practices**				
Bad	0.95 (0.66–1.35)	0.78	0.90 (0.62–1.32)	0.60
Good	1.00 (ref.)		1.00 (ref.)	

Abbreviations: OR, odds ratio; CI, confidence interval; ref, reference. ^a^Adjusted for gender, age, education, place of residence, living status, quarantine status, and information sources. ***p < 0.001 (2-tailed).

**FIGURE 1:**
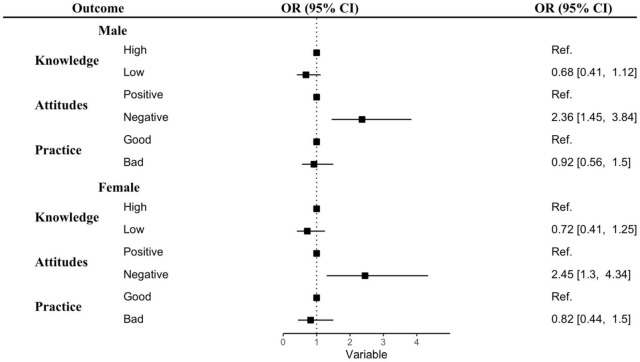
Associations between KAP levels and anxiety during the COVID-19 lockdown by gender. Abbreviations: OR, odds ratio; CI, confidence interval; ref, reference. Adjusted for age, education, place of residence, living status, quarantine status, and information sources.

**FIGURE 2:**
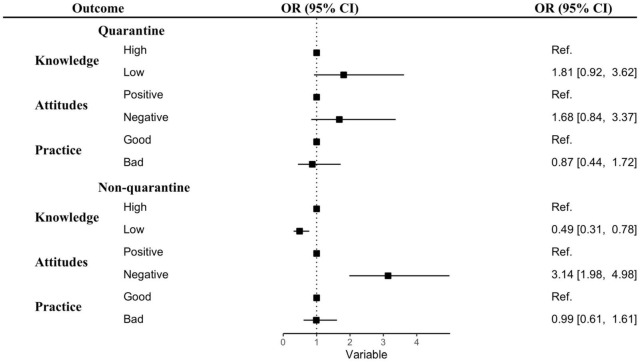
Association between KAP levels and anxiety during the COVID-19 lockdown by quarantine status. Abbreviations: OR, odds ratio; CI, confidence interval; ref, reference. Adjusted for age, gender, education, place of residence, living status, and information sources.

Adjusted associations between KAP and anxiety levels stratified by gender and quarantine status are displayed in Figures 1, 2 and Supplementary Tables 5, 6. Both men and women who had negative attitudes toward COVID-19 were at greater risk of anxiety (Male: OR = 2.36; 95% CI: 1.45–3.84; Female: OR = 2.45; 95% CI: 1.3–4.34; *p* < 0.001) (Figure 1 and Supplementary Table 5). Non-quarantined students showing negative attitudes had over three times the chance of experiencing anxiety (OR = 3.14, 95% CI: 1.98–4.98, *p* < 0.001), and non-quarantined students with a low level of knowledge had half the risk of experiencing anxiety (OR = 0.49, 95% CI: 0.31–0.78, *p* < 0.01). In the case of quarantined students, no significant associations between KAP levels and anxiety were observed (Figure 2 and Supplementary Table 6).

## Discussion

### Summary of the Findings

As one of the densely populated countries in the world, Bangladesh has faced challenges to implementing non-therapeutic measures such as avoiding social gatherings and public transport, wearing masks, washing hands frequently, and other practices. In this catastrophic condition, higher education is one of the worst affected sectors of society. However, past research suggests that adequate knowledge, positive attitudes, and good behavioral practices of students not only supports health and safety but also prevents mental distress. Thus, our study investigated KAP levels toward COVID-19 and associated anxiety in university students during the early stage of the pandemic.

Our results show that approximately half of the students had sufficient levels of knowledge and more than half adhered to COVID-19 precautionary practices. These findings corroborate a previous study with over 10,000 Bangladeshi adults that reported high levels of knowledge regarding COVID-19 preventative behaviors (48). Such findings speak to the effectiveness of delivering massive online public health education during lockdown (49). However, more than half of the students gave incorrect answers regarding the transmission of COVID-19 by food, air, and pets. Also, a few students incorrectly did not think that aches and pains were symptoms of the disease and half of respondents incorrectly associated the occurrence of COVID-19 with nasal congestion. The latter belief could be attributed to mistakes of linking the common fever with cold symptoms (50). The origin of the other incorrect beliefs is unclear but indicates that additional education and research are needed.

Less than half of the students showed negative attitudes toward COVID-19. Mostly, students were unclear about whether their economic conditions and academic careers would be disrupted due to the pandemic. This ambiguity can negatively affect mental health and decision-making ability (51). Similar to other studies (52, 53), half of students realized the importance of social support during the pandemic. Furthermore, nearly one-third of students believed that COVID-19 contributed to societal stigma. These findings highlight the importance of social factors on KAP and mental health among university students during the lockdown, which requires the attention of relevant departments.

Compared to other studies (i.e., 54, 55), our study showed a reduced rate of precautionary behaviors toward COVID-19. Despite having sufficient knowledge, half of students did not adhere to such measures, which was similar to the finding reported by Ferdous et al. (56). Such avoidance can stem from lack of clarity in recommended behavioral measures as well as uncertainty regarding their effectiveness against COVID-19 (57). The percentage of students not adhering to these behaviors was much higher than in some past research (22). One explanation may be the educational gap, which reflects a lack of understanding about public health information. Since our study participants were students, they returned to their homes during lockdown in areas with limited access to information, particularly in rural areas. In addition, people in low-middle income countries, like Bangladesh, generally have poor personal hygiene practices and are less conscious about their health; for instance, only 40% of people have access to facilities to wash their hands with soap and water (58, 59). These factors might contribute to a lack of understanding of COVID-19 protective measures. Although a good knowledge level on infection transmission was found among the students, there was still opportunity to improve these levels. It is widely accepted that a population that is more informed about the disease would adhere to preventative and treatment measures more effectively (56).

Many surveyed students experienced anxiety, including 52% of males and 48% of females. In the early stage of COVID-19, the prevalence of moderate to severe psychiatric symptoms has been documented in several studies (60–63). Prior to the pandemic, the prevalence rate of anxiety was 4.1%, which is approximately five times lower than the present situation (64). Reasons for the higher rates in our sample could be explained by the large share of students (78%) living with their families; one of the reasons for anxiety comes from the fear of spreading the virus between family members through thyself (65). In addition, over 90% of students experiencing anxiety in our sample used social media, such as Twitter and Facebook to update and get information about COVID-19. Past research has found that indirect exposure to mass trauma *via* the media may result in anxiety disorder (66). A recent study conducted in mainland China discovered that increased exposure to social media increased the risk of experiencing anxiety (67).

Our study found that negative attitudes toward COVID-19 were potential risk factors for developing anxiety during the lockdown. In other words, anxiety was comparatively lower among participants who showed positive attitudes toward COVID-19. According to previous research among college students, scholars denoted that positive attitudes were protective against anxiety (OR = 0.822, 95% CI = 0.762–0.887) (21). They mentioned that if people could increase their confidence in resisting COVID-19, it would be advantageous to their mental wellbeing. Another study indicated that young adults intending to gather information about COVID-19 were less likely to develop anxiety (28). As stated in the literature, certain behaviors and responses vary by age and gender (54). One of the reasons for developing anxiety among university students could relate to their academic disruptions. The lockdown caused considerable disruptions that created learning gaps among many students. Such disruptions may have impacted students’ mental health since they were more likely to graduate later than expected (68). To comply with strict precautions, educational institutions had to transfer in-person learning to virtual online classes that created extra burdens among many students (69, 70). Additionally, disruptions in academic activities may have led to uncertainty about future career prospects and therefore increased anxiety (71). Finally, the increasing case counts, lack of proper treatment, absence of available vaccine during the time of this study, media speculation and sensational news could have made students more vulnerable to develop psychological distress during early stage of the COVID-19 pandemic (72–75).

Regarding our stratified analyses, showing negative attitudes was significantly associated with anxiety for males, females, and non-quarantined students. Females indicated stronger effects than males as well. Similar findings were reported elsewhere (21); female students with negative attitudes were more likely to develop anxiety. Usually females are more vulnerable to anxiety and depression because of their social expectations. The situations may be exacerbated during the time of a crisis. An extensive review conducted in 30 countries found a greater prevalence of depression among women (76). Studies have also reported that women are 1.6 times more likely to develop mental disorders than men (77, 78). It is important to mention that women must multitask in household duties while providing caregiving roles. In addition, the closing of educational institutions might have put additional pressures on women. To balance such overloads, women appear to be at particular risk of developing higher disorders (79). When stratified by quarantine status, respondents having negative attitudes and not being in quarantine tended to show higher risks of anxiety. This finding is contrary to an earlier study that showed students in quarantine were more anxious than non-quarantined students (80). This contradictory finding may be explained by the possibility of non-quarantine students being less aware of the impacts of COVID-19 during the early stages of the pandemic. An earlier study (81) reported that two-thirds of student participants had confidence that COVID-19 wouldn’t be a problem in Bangladesh. Despite the government holiday, students could not communicate with their friends in person due to COVID-19 restrictions, which would could have triggered depression and anxiety (82). The prolonged lockdown restricted students from going outdoors and having family outings, and forced to students to remain in the house idly. Consequently, students appear to have more provision to internet access, social media and news exposure and missed out on the salutatory benefits of physical activity and exposure to restorative environments (i.e., green spaces) (83). Furthermore, many news outlets prioritized sensational news and people frequently shared false and negative news that may have sparked mental stress among young adults, particularly students (84).

### Implications

The findings of this study have theoretical and practical implications. Our study is the first of its kind in Bangladeshi university students to examine associations between KAP levels and anxiety. This study therefore expands our understanding about the roles of knowledge, attitudes, and behavioral practices on the mental health of young adults during the COVID-19 pandemic. Even though our study had some limitations, its findings could be relevant for university authorities and policymakers adopting public health interventions in effective and timely manners. Our study suggests that KAPs required to protect students from COVID-19 during the study period were at only moderate levels. Public health education programs should specific target behavioral practices regarding COVID-19 at universities, given the low levels of this dimension of KAPs in our sample. Such programs can be coordinated the Ministry of Education and Ministry of Health and Family Welfare in collaboration with universities. Also, given our notable finding that negative attitudes and less knowledge were associated with anxiety, teachers can play an important role in improving mental health through education and reinforcing positive outlooks toward the COVID-19 situation.

While a wealth of data has been collected on student’s mental health since March 2020, investigations on the psychological and behavioral consequences of lockdowns should continue to be conducted as the pandemic wanes. Simultaneously, interventions should be introduced at universities to alleviate the negative lingering effects of the pandemic on students. Internet based cognitive therapy (CBT) could be an effective way to treat anxiety that works through stress management and relaxation techniques and is convenient for students to complete. Strategies for public policy could also include greater availability of mental health clinicians and psychosocial support interventions. Ultimately, we hope the behavioral data gathered in the current study might serve as a reference for other COVID-19 researchers working on this important and critical area.

### Limitations of the Study

There are some limitations to this research. First, our study was cross-sectional, which was insufficient to explain casual relationships between KAP levels and anxiety. To evaluate these hypothesized causal links, longitudinal investigations may be necessary. Secondly, response biases may have existed in the online and self-reported questionnaires. Without internet connections, respondents could not provide their opinions so our study could not reach these populations. In addition, there could be selection bias due to our use of a non-probability sampling method. Finally, we considered only the early stage of the COVID-19 pandemic, which was a short period of time relative to the entire pandemic. Consequently, our results may not apply to different times of the COVID-19, which means ongoing research should be conducted during the pandemic.

## Conclusion

This is one of the first studies to examine knowledge, attitudes, and behavioral practices knowledge, attitude, and behavioral practice (KAP) levels toward COVID-19 and associated anxiety levels in university students during the first phase of the pandemic. The results provide insights into KAP levels and anxiety rates at this first phase. More than half of students showed high levels of knowledge and good behavioral practices; however, a significant portion of students also held negative attitudes toward COVID-19. Low knowledge levels and negative attitudes were risk factors for anxiety. Consequently, proactive interventions, such as economic and academic security and social support, might be necessary to encourage positive attitudes and psychological welfare. Social support to reduce social stigma is another recommendation. Simultaneously, authentic information sources should be ensured to expand virus-related knowledge and adopt good behavioral practices. The abovementioned suggestions would ultimately support the psychological wellbeing of university students during the ongoing pandemic.

## Data Availability Statement

The raw data supporting the conclusions of this article will be made available by the authors, without undue reservation.

## Ethics Statement

The studies involving human participants were reviewed and approved by Institute of Disaster Management, Khulna University of Engineering & Technology, Khulna, Bangladesh. The patients/participants provided their written informed consent to participate in this study.

## Author Contributions

MP: conceptualization, methodology, formal analysis, writing-original draft, review, and editing. AD and MB: conceptualization and writing-original draft. MHa: conceptualization, data curation, and writing-original draft. SB, MHo, MA, MHB, FS, AP, BZ, SaS, JS, and ShS: writing-review and editing. All authors contributed to the article and approved the submitted version.

## Conflict of Interest

The authors declare that the research was conducted in the absence of any commercial or financial relationships that could be construed as a potential conflict of interest.

## Publisher’s Note

All claims expressed in this article are solely those of the authors and do not necessarily represent those of their affiliated organizations, or those of the publisher, the editors and the reviewers. Any product that may be evaluated in this article, or claim that may be made by its manufacturer, is not guaranteed or endorsed by the publisher.

## Footnotes

1
https://statulator.com/
