# Supplementary material for: Knowledge, Attitudes, and Practices Toward Coronavirus and Associated Anxiety Symptoms Among University Students: A Cross-Sectional Study During the Early Stages of the COVID-19 Pandemic in Bangladesh
Source: Front Psychiatry. 2022 Apr 1;13:856202. doi: 10.3389/fpsyt.2022.856202 (PMC9010605; doi:10.3389/fpsyt.2022.856202)
Supplement: Supplementary file 1 [file Data_Sheet_1.docx]

**Supplementary Information**

**S1 Table.** Knowledge towards COVID-19

| **Statement** | **N (%)** | |
| --- | --- | --- |
|  | **Correct** | **Incorrect** |
| The COVID-19 is an infectious disease | 500 (91.91) | 44 (8.09) |
| **COVID-19 is transmitted, or spread through** |  |  |
| Hand shaking | 528 (97.06) | 16 (2.94) |
| Touching of objects used by an infected person | 524 (96.32) | 20 (3.68) |
| Droplets after coughing or sneezing | 515 (94.67) | 29 (5.33) |
| Face-to-face talk | 421 (77.39) | 123 (22.61) |
| Mosquito bites | 392 (72.06) | 152 (27.94) |
| Faecal-oral transmission | 361 (66.36) | 183 (33.66) |
| Through domestic pet (dog, cat, cow etc.) | 253 (46.51) | 291 (53.49) |
| Food | 209 (38.42) | 335 (61.58) |
| Through Air | 126 (23.16) | 418 (76.84) |
| **Common symptoms of COVID-19 infection** |  |  |
| Fever | 521 (95.77) | 21 (4.23) |
| Difficulty in breathing | 511 (93.93) | 33 (6.07) |
| Sore throat | 500 (91.91) | 44 (8.09) |
| Dry cough | 494 (90.81) | 50 (9.19) |
| Runny nose | 305 (56.07) | 239 (43.93) |
| Aches and Pains | 216 (39.71) | 328 (60.29) |
| Nose Bleeds | 23 (4.23) | 521 (95.77) |
| **Incubation period of COVID-19** |  |  |
| 1-14 days | 476 (87.50) | 68 (12.5) |
| **Individuals at higher risk of COVID-19** |  |  |
| People over 60 years old | 531 (97.61) | 13 (2.39) |
| People with chronic disease | 502 (92.28) | 42 (7.72) |
| Health professionals | 497 (91.36) | 47 (8.64) |
| Young people | 387 (71.14) | 157 (28.86) |
| Pregnant women | 295 (54.23) | 249 (45.77) |
| Children | 260 (47.79) | 284 (52.21) |

**S2 Table.** Attitudes of students towards COVID-19

| **Statement** | **N (%)** | | | | |
| --- | --- | --- | --- | --- | --- |
|  | **Strongly disagree** | **Disagree** | **Undecided** | **Agree** | **Strongly agree** |
| **Negative attitudes** |  |  |  |  |  |
| Worried that your economic condition will be destroyed | 22 (4.04) | 85 (15.63) | 157 (28.86) | 142 (26.10) | 138 (25.37) |
| Worried that your academic routine will be disrupted | 46 (8.46) | 111 (20.40) | 170 (31.25) | 127 (23.35) | 90 (16.54) |
| Infected individuals may face stigma in the society | 48 (8.82) | 140 (25.74) | 175 (32.17) | 133 (24.45) | 48 (8.82) |
| **Positive attitudes** |  |  |  |  |  |
| You believe that the daily life will back to normal soon | 25 (4.60) | 101 (18.57) | 160 (29.41) | 157 (28.86) | 101 (18.57) |
| Social support is necessary to face the difficult situation during COVID-19 | 8 (1.47) | 49 (9.01) | 103 (18.93) | 238 (43.75) | 146 (26.84) |

**S3 Table.** Behavioral practice of students toward COVID-19

| **Statement** | **N (%)** | |
| --- | --- | --- |
|  | **Yes** | **No** |
| Decided to stay at home | 293 (56.86) | 251 (46.14) |
| Avoided touching nose, eyes and mouth | 133 (24.45) | 411 (75.55) |
| Worn a face mask and hand globes | 160 (29.41) | 384 (70.59) |
| Washed hands more frequently with soap and water | 47 (8.64) | 497 (91.36) |
| Used hand sanitizer more regularly | 158 (29.04) | 386 (70.96) |
| Disinfected the home | 206 (37.87) | 338 (62.13) |
| Covered mouth & nose when sneezing or coughing | 118 (21.39) | 426 (78.31) |
| Avoided public transport | 75 (13.79) | 469 (86.21) |
| Avoided social gathering | 39 (7.17) | 505 (92.83) |
| Avoided contact people with fever or other respiratory disease | 137 (25.18) | 407 (74.82) |

**S4 Table.** Full model results of multivariate logistic regression models to determine associations between knowledge, attitude, and practice (KAP) levels toward COVID-19 and anxiety among university students in Bangladesh during the early phases of the pandemic (N=544).

| **KAP Levels** | **Crude model** | | **Fully adjusted model** | |
| --- | --- | --- | --- | --- |
|  | **OR (95% CI)** | **P-value** | **OR (95% CI)** | **P-value** |
| **Knowledge** |  |  |  |  |
| Low | 0.72 (0.51-1.03) | 0.06 | 0.71 (0.50-1.02) | 0.06 |
| High | 1.00 (ref.) |  | 1.00 (ref.) |  |
| **Attitudes** |  |  |  |  |
| Negative | 2.43 (1.71-3.45) | 0.000 | 2.40 (1.66-3.46) | 0.000 |
| Positive | 1.00 (ref.) |  | 1.00 (ref.) |  |
| **Practices** |  |  |  |  |
| Bad | 0.95 (0.66-1.35) | 0.78 | 0.90 (0.62-1.32) | 0.60 |
| Good | 1.00 (ref.) |  | 1.00 (ref.) |  |
| Gender |  |  | 0.72 (0.49-1.03) | .076 |
| Age |  |  | 0.88 (0.46-1.70) | .717 |
| Education |  |  | 0.73 (0.45-1.19) | .214 |
| Residence |  |  | 1.18 (0.71-1.95) | .518 |
| Living |  |  | 0.80 (0.53-1.19) | .283 |
| Quarantine |  |  | 0.91 (0.62-1.34) | .657 |
| Information source |  |  |  |  |
| Government |  |  | 1.09 (0.67-1.75) | .719 |
| International agencies |  |  | 0.95 (0.62-1.43) | .809 |
| Health staff |  |  | 1.20 (0.80-1.79) | .365 |
| Social media |  |  | 0.67 (0.35-1.27) | .223 |
| Traditional media |  |  | 0.91 (0.57-1.45) | .687 |
| Online source |  |  | 1.13 (0.76-1.69) | .535 |

Abbreviations: OR: odds ratio; CI, confidence interval; ref, reference,

**S5 Table.** Association between knowledge, attitudes, and practices toward COVID-19 and anxiety risk stratified by male and female university students in Bangladesh during the early phases of the pandemic (N=544)

| **KAP Levels** | **Male (N=310)** | | **Female (N=234)** | |
| --- | --- | --- | --- | --- |
|  | **Crude model**  **OR (95% CI)** | **Fully adjusted model^a^**  **OR (95% CI)** | **Crude model**  **OR (95% CI)** | **Fully adjusted model^a^**  **OR (95% CI)** |
| **Knowledge** |  |  |  |  |
| Low | 0.74 (0.46-1.18) | 0.68 (0.41-1.12) | 0.71 (0.42-1.22) | 0.72 (0.41-1.25) |
| High | 1.00 (ref) | 1.00 (ref) | 1.00 (ref) | 1.00 (ref) |
| **Attitudes** |  |  |  |  |
| Negative | 2.30 (1.44-3.67)*** | 2.36 (1.45-3.84)** | 2.68 (1.56-4.60)*** | 2.45 (1.3-4.34)** |
| Positive | 1.00 (ref) | 1.00 (ref) | 1.00 (ref) | 1.00 (ref) |
| **Practices** |  |  |  |  |
| Bad | 0.91 (0.57-1.45) | 0.92 (0.56-1.50) | 0.94 (0.53-1.64) | 0.82 (0.44-1.50) |
| Good | 1.00 (ref) | 1.00 (ref) | 1.00 (ref) | 1.00 (ref) |

***p* < .01, ***** *p* < .001 (2-tailed)

Abbreviations: OR, odds ratio; CI, confidence interval; ref, reference.

^a^Adjusted for age, education, place of residence, living status, quarantine status and information sources.

**S6 Table.** Association between knowledge, attitudes, and practices toward COVID-19 and anxiety risk among quarantined and non-quarantined university students in Bangladesh during the early phases of the pandemic (N=544)

| **KAP Levels** | **Quarantine (N=175)** | | **Non-quarantine (N=369)** | |
| --- | --- | --- | --- | --- |
|  | **Crude model**  **OR (95% CI)** | **Fully adjusted model^a^**  **OR (95% CI)** | **Crude model**  **OR (95% CI)** | **Fully adjusted model^a^**  **OR (95% CI)** |
| **Knowledge** |  |  |  |  |
| Low | 1.36 (.74-2.50) | 1.81 (0.91-3.62) | 0.51 (0.33-0.80)** | 0.49 (0.31-0.78)** |
| High | 1.00 (ref) | 1.00 (ref) | 1.00 (ref) | 1.00 (ref) |
| **Attitudes** |  |  |  |  |
| Negative | 1.23 (0.67-2.28) | 1.68 (0.84-3.37) | 3.53 (2.27-5.50)*** | 3.14 (1.98-4.98)*** |
| Positive | 1.00 (ref) | 1.00 (ref) | 1.00 (ref) | 1.00 (ref) |
| **Practices** |  |  |  |  |
| Bad | .75 (0.40-1.39) | 0.87 (0.44-1.72) | 1.11 (0.71-1.74) | 0.99 (0.61-1.61) |
| Good | 1.00 (ref) | 1.00 (ref) | 1.00 (ref) | 1.00 (ref) |

***p* < .01, ***** *p* < .001 (2-tailed)

Abbreviations: OR, odds ratio; CI, confidence interval; ref, reference.

^a^Adjusted for age, gender, education, place of residence, living status, and information sources.
